# Biodegradation of Fumonisins by the Consecutive Action of a Fusion Enzyme

**DOI:** 10.3390/toxins14040266

**Published:** 2022-04-09

**Authors:** Kailin Li, Song Yu, Dianzhen Yu, Huikang Lin, Na Liu, Aibo Wu

**Affiliations:** 1 SIBS-UGENT-SJTU Joint Laboratory of Mycotoxin Research, CAS Key Laboratory of Nutrition, Metabolism and Food Safety, Shanghai Institute of Nutrition and Health, University of Chinese Academy of Sciences, Chinese Academy of Sciences, Shanghai 200031, China; likailin2018@sibs.ac.cn (K.L.); dzyu@sibs.ac.cn (D.Y.); linhuikang2020@sibs.ac.cn (H.L.); liuna@sibs.ac.cn (N.L.); 2 Division of Chemical Toxicity and Safety Assessment, Shanghai Municipal Center for Disease Control and Prevention, Shanghai 200336, China; yusong@scdc.sh.cn

**Keywords:** fumonisin, degradation, fusion enzyme, safety evaluation, control strategy

## Abstract

Fumonisins (FBs) are toxic mycotoxins that commonly exist in food and feed. FBs can induce many aspects of toxicity, leading to adverse effects on human and animal health; therefore, investigating methods to reduce fumonisin contamination is necessary. In our study, we generated a recombinant fusion enzyme called FUMDI by linking the carboxylesterase gene (*fumD*) and the aminotransferase gene (*fumI*) by overlapping polymerase chain reaction (PCR). The fusion enzyme FUMDI was successfully, secretively expressed in the host *Pichia pastoris* (*P. pastoris*) GS115, and its expression was optimized. Our results demonstrated that the fusion enzyme FUMDI had high biodegradation activity of fumonisin B1 (FB1) and other common FBs, such as fumonisin B2 (FB2) and fumonisin B3 (FB3), and almost completely degraded 5 μg/mL of each toxin within 24 h. We also found that FUMDI enzyme and its reaction products had no negative effect on cell viability and did not induce cell apoptosis, oxidative stress, or endoplasmic reticulum (ER) stress in a human gastric epithelial cell line (GES-1). The results indicated that these FBs degradation products cannot have adverse effects in a cell model. In conclusion, a safe and efficient fumonisin-degrading enzyme was discovered, which could be a new a technical method for hazard control of FBs in the future.

## 1. Introduction

Fumonisins (FBs) are toxic mycotoxins mainly produced by *Fusarium verticillioides* (*F. verticillioides*) and *Fusarium proliferatum* (*F. proliferatum*), which host many types of corn [1,2,3]. Fumonisin B1 (FB1) can cause systemic toxicity, including neurotoxicity, hepatotoxicity, nephrotoxicity, mammalian cytotoxicity, immunotoxicity, and carcinogenicity [2,4,5,6,7]. Furthermore, FB2 and FB3 cause toxicological and histopathological effects mimicking the effects of FB1 [8,9].

Fumonisin contamination is widespread throughout the world, mainly contaminating corn and its products. The ratio of positive fumonisin-contaminated samples in random samples was up to 74% in Argentina [10]. Furthermore, total fumonisin levels ranged from 0.28 to 15 mg/kg with a mean of 1.54 ± 0.12 mg/kg, and 20% of the prepared feed samples randomly collected from 133 commercial poultry farms, 76 feed processors and 8 feed vendors of Ghana were higher than the FAO/WHO maximum tolerable daily intake limit of 2 mg/kg [11]. Studies also revealed that 47% of Mexican men and 30% of Mexican women might exceed the provisional tolerable daily intake (PMTDI) of 2 µg/kg bw/day for FBs [12]. *Aspergillus niger*, intended for use in the food industry in China, can cause dynamic FB2 production to reduce human exposure to FBs [13]. In view of the toxicity of FBs, it is necessary to take measures to reduce their contamination levels, thus decreasing health hazards to humans and animals [14,15].

**Figure 1:**
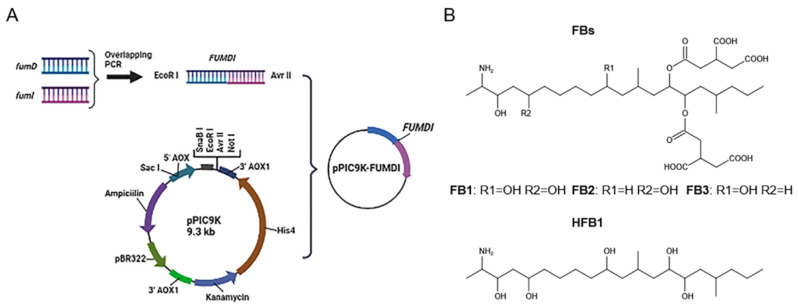
Flowchart for the construction of fusion genes and recombinant plasmids and structural formulas of fumonisins (FBs). (**A**) The flow chart of the fusion enzyme FUMDI expression vector construction. (**B**) Chemical structures of FBs and HFB1.

The removal strategies of FBs mainly include physical, chemical, and biodegradation methods [16,17]. Physical and chemical methods have shortcomings such as low removal efficiency, easy damage to product quality, and introduction of new toxic substances [17]. Similarly, when fumonisin is degraded by microorganisms, specific environmental conditions are required for microbial growth, which greatly limits the scope of microbial degradation method application. In addition, microorganisms also secrete various unknown substances, which pose certain health safety risks. Different from the above methods, enzymes can specifically remove the toxic group of mycotoxins, converting them to less toxic or nontoxic forms without producing impurities. At present, enzymatic degradation is considered an efficient, specific, safe, and environmentally friendly high-quality method [18]. The tricarboxylic acid group (-TCA) and amino group (-NH2) of FBs (Figure 1) can competitively inhibit sphingosine N-acetyltransferase activity during sphingolipid metabolism and block the signaling of complex sphingolipids as second messengers to affect cell proliferation, differentiation, and apoptosis. Therefore, the -TCA and -NH2 groups are considered the toxic groups of FBs [19]. Currently, the applied FB1 degradation enzymes are mainly carboxylesterases, which can remove the -TCA of FB1 and then generate hydrolyzed fumonisin B1 (HFB1) (Figure 1) [18]. Although this process reduces the toxicity of FB1, the toxicity of HFB1 with amino groups has been gradually discovered. Studies have reported that HFB1 was more bioavailable than FB1 on intestinal cells and could competitively inhibit the enzyme ceramide synthase and form acetylated products, and its acetylated product N-acyl HFB1 was more cytotoxic than HFB1 [20,21,22,23]. Yu et al. found that HFB1 could significantly reduce body weight and disrupt gut microbial balance in broilers [24].

Degrading enzymes are usually produced by fermentation using prokaryotic expression systems or eukaryotic expression systems [25]. Prokaryotic expression systems often have drawbacks, such as insoluble protein expression and protein modification, which seriously affect the activity and yield of fermentation products. However, eukaryotic expression systems can avoid these shortcomings and are currently considered to be efficient in vitro protein expression systems [26]. *Pichia pastoris* (*P. pastoris*) GS115 is a widely used system for the large expression of heterologous proteins [27,28]. In our study, a fusion enzyme of carboxylesterase and aminotransferase for the subsequent degradation of FB1 was designed, constructed, and expressed in a safe manner. The fusion enzyme was cloned into the pPIC9K vector and successfully, secretively expressed in the host *Pichia pastoris* GS115 and named FUMDI. The degradation efficiency of the fusion enzyme FUMDI was optimized, and the safety was evaluated in GES-1 cells by cell viability, apoptosis, oxidative stress, and ER stress assessments in vitro. Our study provides a safe and convenient way to control FB contamination in food products.

## 2. Results

### 2.1. Cloning of the Fusion Gene FUMDI and Construction of Recombinant Plasmids

**Figure 2:**
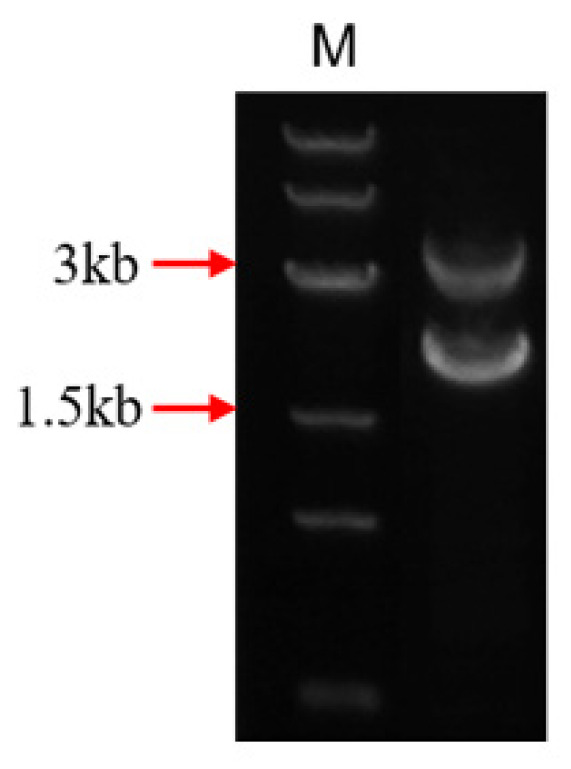
Agarose gel electrophoresis identification of the FUMDI-pPIC9K vector.

We linked the carboxylesterase gene (*fumD*) and the aminotransferase gene (*fumI*) nucleotide sequences using an overlapping double-polymerase chain reaction (PCR) and cloned the fusion sequence into the expression vector pPIC9K to generate the recombinant plasmid pPIC9K-FUMDI (Figure 1). The recombinant plasmid was transformed into *P. pastoris *GS115-competent cells and verified by PCR using the primers p9k-F and p9k-R. As the results show in Figure 2, the band size was 2.9 kb as expected as a theoretical value, indicating that the fusion gene sequence was cloned into the pPIC9K vector by PCR.

### 2.2. Expression and Purification of the FUMDI Enzyme

**Figure 3:**
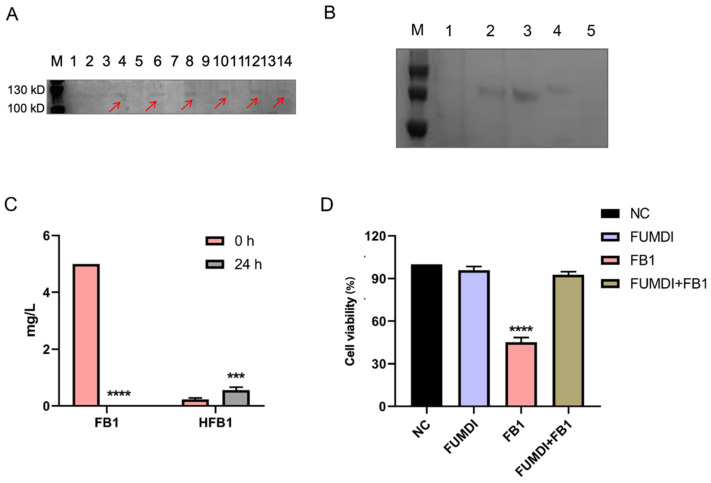
Fusion enzyme FUMDI expression. (**A**) SDS–PAGE gel staining of pPIC9K and pPIC9K-FUMDI at different timepoints (Line 1, 2: 24 h; Line 3, 4: 36 h; Line 5, 6: 48 h; Line 7, 8: 60 h; Line 9, 10: 72 h; Line 11, 12: 84 h; Line 13, 14: 96 h), the red arrow indicates the estimated protein band. (**B**) SDS–PAGE gel staining of purified FUMDI enzyme, where M: protein marker, L1: FUMDI enzyme precipitation using 70% ammonium sulfate, L2: FUMDI enzyme precipitation using 80% ammonium sulfate, L3: FUMDI enzyme precipitation using 90% ammonium sulfate, L4: FUMDI enzyme precipitation using supersaturated ammonium sulfate, and L5: protein expression control in *Pichia pastoris* (*P. pastoris*) GS115 with the pPIC9K plasmid. (**C**) FB1 degradation of 5.0 μg/mL by FUMDI enzyme. (**D**) Effect of purified FUMDI enzyme and FUMDI final product on GES-1 cells at 48 h. The values are the mean ± SD of three independent experiments. NC, negative control, *** and **** indicates a significant difference between treatment groups (**C**) or treatment groups and control (**D**) at *p* < 0.001 and *p* < 0.0001, respectively.

The pPIC9K vector was used for secretory expression, and the AOX1 promoter region helped induce *P. pastoris* GS115 expression (Figure 1). To induce protein expression, *P. pastoris* GS115 (pPIC9K-FUMDI) cells were transferred into BMMY induction medium and cultivated at 30 °C at 300 rpm/min after the OD600 of the cultures reached 2.0–6.0 in BMGY. To explore the optimal induction expression time of the FUMDI enzyme, we detected the expression of FUMDI at different time points. In Figure 3A, the fusion-induced protein migrated with an apparent molecular weight on sodium dodecyl sulfate–polyacrylamide gel electrophoresis (SDS–PAGE). Gel staining showed that 110 kDa, which was close to the estimated protein size of FUMDI, was most highly expressed when the induction time was 60 h (Figure 3A). The results verified that the fusion protein FUMDI successfully achieved soluble expression in *P. pastoris* GS115. For purification of the fusion enzyme FUMDI, protein supernatant was precipitated by different concentrations of ammonium sulfate (Figure 3B). According to Figure 3B, FUMDI could not be precipitated when the ammonium sulfate concentration was less than 70%.

### 2.3. FB1 Degradation by the FUMDI Enzyme

Fusion enzyme FUMDI supernatant was mixed with 5 μg/mL FB1 in reaction buffer at 25 °C for 24 h. Then we analyzed the changes in the concentration of FB1 and HFB1 using LC–MS/MS (Table 1). Firstly, FB1 was converted to HFB1 by carboxylesterase of FUMDI. Secondly, HFB1 was converted to 2-keto-HFB1 by aminotransferase of FUMDI. After 24 h, FB1 was almost completely degraded (Figure 3C). In Figure 3C, it can be seen that FUMDI completely degraded FB1 in 24 h, and only a small fraction of the intermediate HFB1 remained, indicating that FUMDI can consecutively degrade FB1 within 24 h. We also used an empty vector expression product and buffered it with FB1 toxin; however, we observed that the empty vector expression product and reaction buffer had no degradation activity. Accordingly, the fusion enzyme FUMDI can consecutively degrade FB1 to nontoxic products.

### 2.4. Reverse Effect of Fusion Enzyme FUMDI on FB1-Induced Cell Viability Decrease

To ensure that the FUMDI enzyme and the final product had no harmful effect on the human body, the human gastric epithelial cell line (GES-1) was chosen for the cell viability test. We used 100 μg of the purified and concentrated FUMDI enzyme for incubation with FB1 toxin. In Figure 3D, the consequences of FB1, purified FUMDI enzyme, and FB1 degradation products on GES-1 cells after 48 h of incubation performed using the Cell Counting Kit-8 (CCK-8) test are shown. In our previous study, cell viability was significantly decreased when cells were treated with 10 μM FB1 [9]. Therefore, 10 μM was selected as the FB experimental dose in the subsequent test. After 48 h of incubation, the cell treatment group with 10 μM FB1 exhibited a remarkable decline in cell viability (Figure 3D). The impact of the FUMDI enzyme and the final degradation products of FB1 showed that they had no significant cell death effect in the in vitro cell cytotoxicity study (Figure 3D). Therefore, we conclude that FUMDI can consecutively degrade FB1 and HFB1 in vitro, and cell viability inhibition and cell injury caused by FB1 can therefore be avoided by the FUMDI enzyme.

### 2.5. Effect of Fusion Enzyme and Its Final Products on Cell Apoptosis, Oxidative Stress, and Endoplasmic Reticulum (ER) Stress

**Figure 4:**
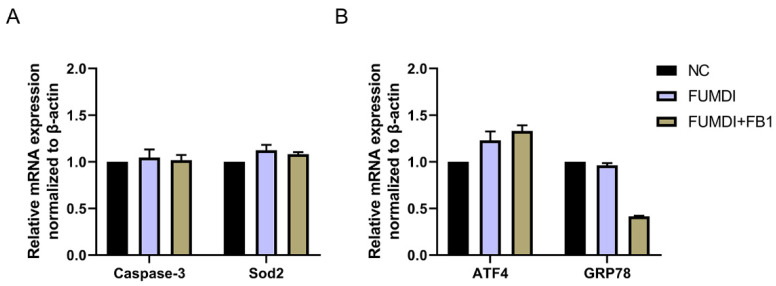
Effect of fusion FUMDI enzyme final products on apoptosis markers (**A**) and ER stress markers in GES-1 cells (**B**).

As exogenous proteins, enzymes may affect the normal growth and proliferation of cells. To evaluate the cell safety of FUMDI and its degradation products, we determined the expression level of genes related to apoptosis, oxidative stress, and ER stress in GES-1 cells using qRT–PCR. After treatment with 100 μg/L FUMDI enzyme and FUMDI enzymatic degradation final products, the expression levels of Caspase-3 and Sod2 in GES-1 cells were nearly unchanged, indicating that FUMDI and its FB1 degradation products did not cause cell apoptosis or oxidative stress (Figure 4A). The expression of ATF4 was slightly but not significantly upregulated in treated cells (Figure 4B). The expression of GRP78 was unexpectedly downregulated in the FUMDI product-treated cells, indicating that the degradation process can alleviate ER stress under normal conditions (Figure 4B). The results demonstrated that FUMDI and its FB1 degradation products cannot have a negative effect on cell survival and proliferation.

### 2.6. Fusion Enzyme FUMDI Degradation Efficiency on Other FBs

**Figure 5:**
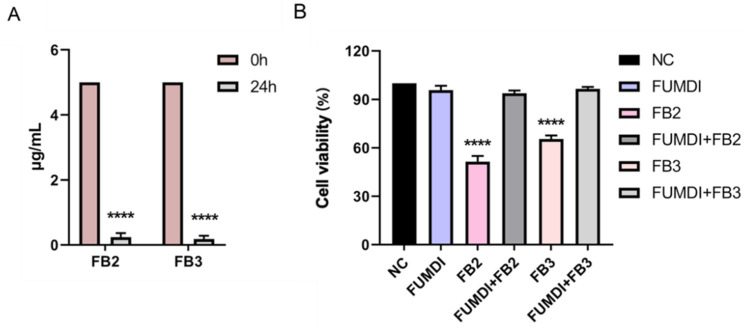
Degradation efficiency of FUMDI on other FBs (**A**) and safety evaluation of degradation products in GES-1 cells (**B**). The values are the mean ± SD of three independent experiments. NC, negative control, **** indicate a significant difference between treatment groups (**A**) or treatment groups and control (**B**) at *p* < 0.0001, respectively.

FB2 and FB3 are other main forms of FBs and usually coexist with FB1 [29]. Although FB1 is more prevalent, FB2 has been found at higher levels than FB1 in some reports [10]. Moreover, there has been a remarkable increase in dietary exposure to fumonisin B3 (FB3) over the last decade, which warrants further attention [29]. FB2 and FB3 are less toxic than FB1; however, they can also cause a decrease in cell viability and an increase in membrane leakage, cell death, and the induction of the expression of markers for endoplasmic reticulum stress [9]. Accordingly, we also evaluated the efficiency of the fusion enzyme FUMDI in degrading FB2 and FB3 and verified the protection of FUMDI against cellular damage caused by FB2 and FB3. After 48 h of incubation with the fusion enzyme FUMDI in vitro, 5 μg/mL FB2/FB3 was almost totally eliminated (Figure 5A). Then we treated GES-1 cells with FUMDI-purified enzyme solution and the final degradation products of FB2/FB3 by the fusion enzyme FUMDI. As expected, we found that the fusion enzyme FUMDI had no significant effect on cell viability and avoided the toxicity of FB2/FB3 to GES-1 cells (Figure 5B).

## 3. Discussion

Worldwide mycotoxin contamination has a broad and serious impact on animal and human health and causes great financial losses, accounting for billions of dollars annually. The application of preharvest and postharvest strategies, including chemical or physical methods, are not sufficiently efficient and somewhat negatively impact the quality of food products. Biological transformation is considered as the most promising but challenging way to reduce mycotoxin accumulation. Although some microorganisms can degrade mycotoxins, only a few enzymes can be maturely applied for this activity [2,17,30].

**Figure 6:**
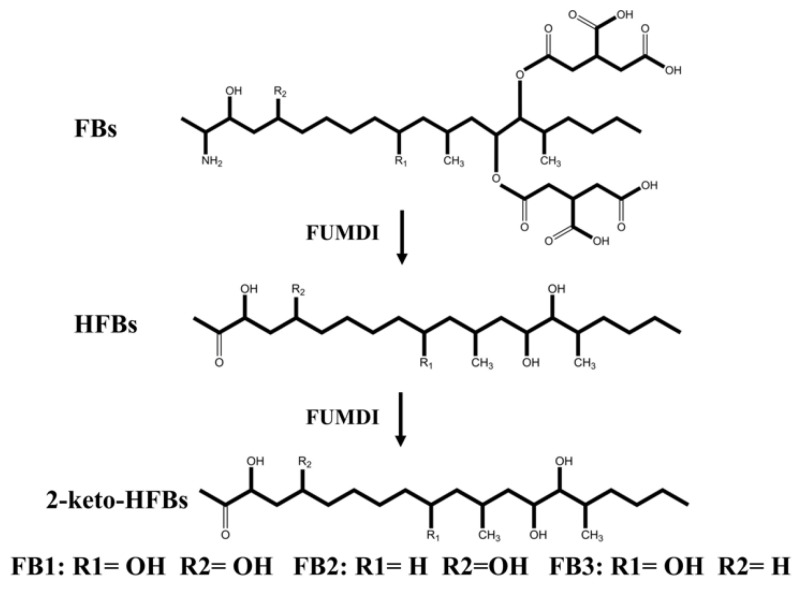
The biodegradation process of fumonisin by FUMDI enzyme.

Fumonisin is a secondary metabolite produced by *Fusarium verticillioides* (*F. verticillioides*) and *Fusarium proliferatum* (*F. proliferatum*). Twenty-eight species have been identified so far, of which FB1 is the most prevalent and the most toxic toxin [2]. FB1 causes hepatotoxicity, neurotoxicity, nephrotoxicity, immunotoxicity, reproductive toxicity, and embryotoxicity and affects the sensory characteristics of feed, leading to a decrease in food intake and production performance and causing large economic losses [31,32]. Enzymatic treatment is an attractive method for FB1 detoxification. Some carboxylesterases and peroxidases can reduce FB1 toxicity [17,18]. FumD is currently commonly used for FB1 detoxification in raw feed materials due to its efficiency in degrading FB1 to its less toxic metabolite HFB1 and alleviating the effects of FB1 on animals [18,33,34,35]. Although HFB1 was usually regarded as a low-toxicity degradation product of FB1, it was found to able to reduce growth performance and disrupt gut microbial balance in broilers and was able to be converted to a more toxic acetylated product of N-acyl HFB1 [20,21]. Therefore, the transformation of FB1 to HFB1 is not a complete detoxification process. In our study, we constructed a secretory expression vector to obtain the fusion enzyme FUMDI, which can degrade FB1 and HFB1 via a continuous reaction (Figure 6) and evaluated the cell safety of the enzyme and its degradation products.

Apoptosis is a highly regulated process of cell death, and both external and internal stimuli, coupled with extrinsic and intrinsic apoptosis pathways, can initiate apoptosis [36]. To ensure the safety of the FUMDI enzyme and its FB1 degradation products, we quantified the gene expression of the common apoptosis marker caspase-3 and the oxidative stress marker Sod2. The qRT–PCR results verified that the enzyme and its products did not stimulate cell apoptosis (Figure 5A). ER stress activation can induce apoptosis and our previous results showed that FB1 is cytotoxic and induces ER stress [37]. To ensure that GES-1 cell exposure to FUMDI and its products does not cause ER stress, we analyzed the expression of ER stress markers by qRT–PCR. Figure 5B indicates a slight upregulation of ATF4, but not a significant activation of ER stress. Furthermore, the expression of GRP78 was even downregulated when cells were treated with FB1 degradation products, which may indicate that pyridoxal phosphate in the enzyme reaction system may alleviate ER stress to some degree. These findings suggest that the FUMDI fusion enzyme and its FB1 degradation products are nontoxic to GES-1 cells.

Although considerable research has focused on new methods to prevent and attenuate FB1-induced toxic consequences, there are few detoxification methods for FB2 and FB3, which have similar toxicity to FB1 [38]. Our results revealed that the fusion enzyme FUMDI can efficiently degrade FB2 and FB3, and no negative effects on cell viability from their degradation products were observed in our study. This enzyme may be a promising method to simultaneously control FB1, FB2, and FB3 contamination in food and feed products (Figure 6).

## 4. Conclusions

In this study, we constructed an expression vector of fumonisin-fusion-degrading enzyme FUMDI with genetic engineering technology, and successfully achieved secretory expression in the host Pichia pastoris GS115. At 25 °C and pH 7, the fusion enzyme almost completely degraded 5 mg/mL FB1 within 24 h, converting FB1 to 2-keto-HFB1 in a buffer system. The results of cytotoxicity evaluation showed that the enzyme and its degradation products did not cause harm to cells and had good safety. In addition, this fusion enzyme also degraded FB2 and FB3. In conclusion, this study developed an efficient and safe fumonisin-degrading enzyme, which has good solubility and is suitable for purification and industrial production. In the future, we will explore the degradation effect and suitable degradation conditions of degrading enzymes in real samples (such as grains). This is a promising approach to control fumonisin contamination to reduce human exposure to mycotoxins.

## 5. Materials and Methods

### 5.1. Chemicals and Reaction System

Fumonisin B1 and B2 were purchased from Abcam (Cambridge, MA, USA). Fumonisin B3 was purchased from Cayman (Ann Arbor, MI, USA). Hydrolyzed fumonisin B1 was obtained from Romer (Beijing, China). Ultrapure water (18.2 MΩ·cm) used in our experiments was supplied by Millipore (Bedford, IN, USA). Restriction enzymes, T4 DNA ligase, and plasmid extraction kits were purchased from Takara Biomedical Technology (Beijing, China). Protein markers, protein-loading buffers, and SDS gel stain washing buffers were purchased from Epizyme (Shanghai, China). Methanol, formic acid, and acetonitrile were purchased from Anpel (Shanghai, China). All other reagents and chemicals were of analytical grade.

### 5.2. Plasmids, Strains, Cultural Conditions, and DNA Manipulations

The coding genes for the enzyme FUMDI were synthesized and cloned into DH5α (pPIC9K-FUMDI) using pEASY^®^-T1 Cloning Kit (TransGen, Beijing, China). The expression host *P. pastoris* GS115 and the secretory expression vector pPIC9K were preserved in this laboratory. The plasmid pPIC9K-FUMDI and recombinant strain *P. pastoris* GS115 (pPIC9K-FUMDI) were constructed in this work.

For cultivation of *P. pastoris* GS115 (pPIC9K-FUMDI), the strain was grown in YPD medium (yeast extract 1%, peptone 2%, glucose 2%, pH 6.3) and BMGY medium (1.0% yeast extract, 2.0% peptone, 1.34% yeast nitrogen base W/O, 4 × 10–5% biotin, 1 M potassium phosphate buffer pH 6, and 1% glycerol) at 30 °C and 300 rpm/min for 16 to 18 h. For induction expression of *P. pastoris* GS115 (pPIC9K-FUMDI), BMMY medium (1.0% yeast extract, 2.0% peptone, 1.34% yeast nitrogen base W/O, 4 × 10–5% biotin, 1 M potassium phosphate buffer pH 6, and 1% methanol) was used, and the strain was cultivated at 30 °C and 300 rpm/min.

### 5.3. Construction of Fusion Genes and Recombinant Plasmids

Nucleotide sequences of the carboxylesterase gene (*fumD*) and the aminotransferase gene (*fumI*) of *Sphingopyxis* macrogoltabida strain MTA144 fumonisin catabolism gene cluster (NCBI: FJ426269.1) were extracted as templates. The primers fumD-F and fumD-R, fumI-F and fumI-R were used for PCR amplification of the two genes, respectively. The two PCR products were purified. These PCR results were mixed as templates, and the primers fumD-F and fumI-R were used for overlapping PCR to obtain the fusion genes. The fusion genes obtained were then cloned into the expression vector pPIC9K to generate the recombinant plasmid pPIC9K-FUMDI. The recombinant plasmid from the positive clones was purified, verified by DNA sequencing, and then transformed *P. pastoris* GS115. Positive transformants will show the 2.2 kb wild-type AOX1 band and the 2.9 kb product containing the target gene and the 492 bp flanking sequence of the AOX1 gene in the pPIC9k vector. The primers used for plasmid construction and verification are listed in Table S1.

### 5.4. Expression of Fusion Enzyme FUMDI in Recombinant P. pastoris GS115

The recombinant *P. pastoris* GS115 (pPIC9K-FUMDI) was cultivated in BMGY medium at 30 °C for approximately 16 to 18 h at a speed of 300 rpm/min. When the OD_600_ of the cultures reached 2.0–6.0, the cells were transferred into BMMY induction medium and cultivated at 30 °C at 300 rpm/min for 24, 36, 48, 60, 72, 84, and 96 h for expression of the fusion enzyme. Every 24 h, 1% methanol was added. The supernatants of these cultures at different time points were collected by centrifugation, and the protein samples were concentrated with different concentrations of ammonium sulfate and 50-kDa ultrafiltration tubes according to the manufacturer’s instructions. The concentrated solution samples containing the enzyme were used for subsequent cell assay analysis. Proteins were analyzed and identified by SDS–PAGE.

### 5.5. Degradation of FB1 and HFB1 by FUMDI

An aliquot of 900 μL of crude fusion enzyme supernatant was mixed with 100 μL 10 × buffer (200 mM Tris–HCl, pH 8, 1 mg/mL BSA, 200 μM pyridoxal phosphate, 30 mM pyruvate) and FB1, and the final nominal FB1 concentration was 5.0 μg/mL. For FB1 degradation in the reaction system, the reaction temperature of 25 °C at pH 7, was investigated, followed by 95 °C for 5 min to terminate the reaction. The FB2 and FB3 degradation reaction conditions were the same as those for FB1. All experiments were performed in triplicate.

### 5.6. Mycotoxin Extraction and LC–MS/MS Analysis

Samples were extracted by mixing flour with 10 mL of acetonitrile: water: formic acid (840:159:1 *v*/*v*). Then, they were centrifuged at 4000 rpm/min for 10 min and filtered through a 0.22 μm nylon filter. From the filtered solution, 1 mL was taken and stored in sampler vials at −20 °C until LC–MS/MS analysis. 

Mycotoxins were determined using an Accela 1250 UPLC system (Thermo Fisher Scientific, San Jose, CA, USA) coupled to a TSQ VantageTM (Thermo Fisher Scientific, San Jose, CA, USA) triple-stage quadrupole mass spectrometer. The chromatographic column used in this method was an AgilentExtend-C18 (100 mm × 4.6 mm, 3.5 μm) column with a flow rate of 0.35 mL/min at 30 °C. The injection volume was 10 μL. For FBs, the mobile phase consisted of water containing 0.1% formic acid (A) and methanol containing 0.1% formic acid (B). The gradient was as follows: 0 min 29% B, 3 min 74% B, 5 min 74% B, 5.2 min 100% B, 5.7 min 100% B, 5.8 min 29% B, and 10 min 24% B. Mass spectrometry analysis was performed in positive ionization mode (ESI + 3.5 kV) using selected reaction monitoring (SRM). For MS/MS analysis, the optimized conditions were set as follows: vaporizer temperature at 300 °C; capillary temperature at 300 °C; sheath gas pressure at 10 psi; aux gas pressure at 15 psi. Data analysis was performed with XcaliburTM software 4.1 (Thermo Fisher Scientific, San Jose, CA, USA, 2011).

### 5.7. In Vitro Cell Cytotoxicity Assay

#### 5.7.1. Measurement of Cell Viability 

Dulbecco’s Modified Eagle Medium with high glucose (H-DMEM) was purchased from HyClone (Logan, UT, USA). The human gastric epithelial cell line (GES-1) was obtained from Beijing Beina Chuanglian Biotechnology Institute (Beijing, China) and cultured in H-DMEM (HyClone) with 10% fetal bovine serum (Invitrogen) and penicillin–streptomycin–amphotericin B (BioInd, Cromwell, CT, USA). When the cells reached 80%, the medium was changed after washing twice with phosphate buffered saline (PBS), and trypsin containing 0.25% EDTA was applied for 1 min until most of the cells detached. After centrifugation for 5 min at 1000 rpm/min, the collected cells were dissolved in DMEM and plated in a 96-well plate (Thermo Fisher Scientific, Inc., Beijing, China) with 10,000 cells per well. After growth for 24 h, solutions of FB, HFB1, purified FUMDI enzyme, and degradation final products were added to the cells at concentrations of 10 μM and 10% (*v*/*v*) enzyme and enzyme degradation products and incubated for 48 h. Afterwards, the viability of the cells was measured using Cell Counting Kit-8 (CCK-8) (Dojingdo Laboratories, Kumamoto, Japan) according to the company’s instructions.

#### 5.7.2. Total RNA Extraction and qRT–PCR Determination of Genes Related to Apoptosis and ER Stress

The GES-1 cells were cultured in a 6-well plate at a concentration of 60,000 cells per well and incubated for 24 h. Following the incubation, the culture medium was replaced with new medium containing 10 μM FB1, 100 μg/mL purified DI enzyme, and 0.1% FB1 final degradation products (initial FB1 reaction concentration: 100 μM) for another 48 h, where buffer (final concentration: 0.1%) as the negative control acted as the well media. After 48 h, TRIzol reagent (Invitrogen, Waltham, MA, USA) was used for total RNA extraction. One μg of mRNA was converted to cDNA by a PrimeScript synthesis kit (Takara, Beijing, China) and amplified by quantitative real-time polymerase chain reaction (qRT–PCR) using a TB Green Premix Ex Taq II (Takara, Beijing, China). The 2^−ΔΔCt^ method was used to relatively quantify the mRNA expression of apoptosis and oxidative stress-related genes (Caspase3, Sod2) and ER stress-related genes (ATF4, GRP78), and β-actin was used as the internal reference gene. The sequences of the specific primers used are shown in Table S1. Quantitative reverse transcription PCR was performed by a QuantStudio Real-Time PCR Q6 thermal cycler (Applied Biosystems, Foster City, CA, USA).

### 5.8. Statistical Analysis

The mycotoxin concentration was assessed by analysis of variance (ANOVA). Post hoc Tukey’s test was used to evaluate progressive changes between groups. A value of *p* < 0.001 was considered statistically significant. Statistical analyses were performed with SPSS 22.0 (SPSS Inc., Chicago, IL, USA).

## Figures and Tables

**Table 1:** MS/MS parameters for detected mycotoxins in SRM mode.

Mycotoxin	Precursor Ion (*m*/*z*)	Retention Time (min)	Product Ion (*m*/*z*)	Collision Energy (eV)
FB1	722.4	5.16	352.3 * 334.0 704.5	40 34 18
FB2	706.3	6.32	336.5 * 354.5 318.3	29 36 35
FB3	706.3	5.63	336.5 * 354.5 318.3	29 36 35
HFB1	406.3	4.94	370.6 * 388.6	17 15

* Quantification product ion.

## Data Availability

The data that support the findings of this study are available from the corresponding author upon reasonable request.

## Supplementary Materials

The following supporting information can be downloaded at: https://www.mdpi.com/article/10.3390/toxins14040266/s1, Table S1: Primers sequences used for RT-qPCR amplification.

## Abbreviations

CCK-8	Cell Counting Kit-8
*F. verticillioides*	*Fusarium verticillioides*
*F. proliferatum*	*Fusarium proliferatum*
*P. pastoris*	*Pichia pastoris*
ER	endoplasmic reticulum
FBs	fumonisin Bs
FB1	Fumonisin B1
FB2	Fumonisin B2
FB3	Fumonisin B3
GES-1	human gastric epithelial cell line
HFBs	Hydrolyzed Fumonisin Bs
HFB1	Hydrolyzed Fumonisin B1
LC–MS/MS	liquid chromatography–tandem mass spectrometry
PCR	double-polymerase chain reaction
