# Supplementary material for: Biodegradation of Fumonisins by the Consecutive Action of a Fusion Enzyme
Source: Toxins (Basel). 2022 Apr 9;14(4):266. doi: 10.3390/toxins14040266 (PMC9025740; doi:10.3390/toxins14040266)
Supplement: Supplementary file 1 [file toxins-14-00266-s001.zip › toxins-1654589-supplementary.pdf]

toxins

Article

Biodegradation of Fumonisins by the Consecutive Action of a Fusion Enzyme

Kailin Li, Song Yu, Dianzhen Yu, Huikang Lin, Na Liu and Aibo Wu

Table S1. Primers sequences used for RT-qPCR amplification.

Gene name	Primers sequences
	Complementation primers
fumD	F: GCGAATTCATGCATCATCACCATCACCATAAAGAACACCAATGTAGA<br>R: GTCTCGTACCATTGGCCTTAGATGGTTGACATGCTT
fumI	F: AAGCATGTCAACCATCTAAGGCCAATGGTACGAGAC<br>R: GCCCTAGGTCAAGCACCAGCAAGTTGCATC
M13	F: CGCCAGGGTTTTCCCAGTCACGAC<br>R: GAGCGGATAACAATTTCACACAGG
pPIC9K	F: TGGTGGGAATACTGCTGATAG<br>R: CCCAACTTGAACTGAGGAAC'
	qRT-PCR primers
β-Actin	F: CCTGGCACCCAGCACAAT<br>R: GGGCCGGACTCGTCATAC
Caspase-3	F: GCTGAGCTGCCTGTAACTTGAGAG<br>R: CATGGCTCTGCCTTCATGGAACAG
Sod2	F: AACCTCACATCAACGCGCAGATC<br>R: CTCCTGGTACTTCTCCTCGGTGAC
GRP78	F: TTGTTCTTGTTGGTGGCTCGACTC<br>R: GACAGCAGCACCATACGCTACAG
ATF4	F: GTTCCTGCTGCCTGGTGTCTTG<br>R: GCACGCCACCTTCTCAATTCATTC
